# Comparative Evaluation of shear Bond Strength of universal Dental Adhesives -An *in vitro* study

**DOI:** 10.4317/jced.53816

**Published:** 2017-07-01

**Authors:** Arun Jayasheel, Nandini Niranjan, Hemanthkumar Pamidi, Mayuri B. Suryakanth

**Affiliations:** 1 Reader, Bapuji Dental College & Hospital, Davangere; 2 Post graduate, Bapuji Dental College & Hospital, Davangere; 3 Dental Health officer, Government hospital, Bhadravathi, karnataka

## Abstract

**Background:**

Patient demand for tooth colored restorations and desire for minimally invasive restorations have made composites an indispensable part of the restorative process. An important factor affecting the intra-oral performance of composite restorations is bonding.

**Material and Methods:**

Ninty six freshly extracted molar teeth were collected and occlusal 3mm is removed using a diamond disc to expose dentine. Following with samples were divided in to two main groups (self-etch & total etch). Each main group is again sub divided in to three groups each according to bonding agent used (Tetric N- Bond Universal, Single Bond Universal, Tetric N Bond Total etch in total etch group and Clear Fill SE in self etch group). Following which bonding protocol is followed according to manufacture instructions, a composite buildup of 2x3 mm is done on each specimen and then specimen were subjected to shear bond test under universal testing machine. All the readings were noted and subjected to statistical analysis using One way ANOVA and Tukey’s posthoc test.

**Results:**

It showed that there is no significant difference among the groups in both self-etch and total etch modes.

**Conclusions:**

It can be concluded that application of an etching step prior to Universal Adhesives significantly improves their dentine penetration pattern, although this does not affect their mean SBS. The bond strength values of the TBU regardless of application mode were comparable to SBU making them reliable for working under different clinical conditions.

** Key words:**Dentine bonding agents, self-etch mode, total etch mode, shear bond strength.

## Introduction

Patient claim for tooth colored restorations and desire for minimally invasive restorations have made composites an indispensable part of the restorative process (1). An important factor affecting the intra-oral performance of composite restorations is bonding (2).

Modern adhesive dentistry offers significant advantages; for example, it allows conservation of hard tissue and makes possible for effective and efficient restoration. The goal in adhesive dentistry is to achieve an adequately strong bonding of the restorative resin to the tooth structure so that there is optimum retention, reduced microleakage and, hence, superior color stability and clinical longevity of the restoration (3).

The main challenge for a dental adhesive is the ability to bond effectively to substrates of different nature. Bonding to enamel is reliable and durable, basically requires etching with an acid, commonly 30% to 40% of phosphoric acid prior to application of a fluid adhesive resin. The acid etch step results in selective demineralization of prismatic and interprismatic enamel. Simple micromechanical interlocking is then obtained upon in situ polymerization of resin in the acid induced porosity. The first bonding protocol that revealed a clinically acceptable outcome involved the complete removal of the smear layer by a ‘total-etch’ and now better termed ‘etch-and rinse’ approach. These multi-step dental adhesives have been marketed since the early 1990s and can still today be considered as ‘gold-standard’ adhesives (4).

The market-induced demand for simplified adhesive procedures has rapidly led to the development of the self-etch adhesives which follow a trend towards simplification. The self-etching primer and adhesives were developed in order to avoid the adverse effects of over etching and under/over priming. One advantage with the use of self-etch adhesives is that prior removal of the smear layer and smear plugs is not required as these systems are capable of etching the tooth surface, while simultaneously preparing it for adhesion (5).

Manufacturers are constantly introducing new adhesive systems with claims of Simplicity in use,improvement in their composition and ability to bond to tooth structure. Scientists and researchers feel the obligation to substantiate this claims. Previous studies have shown that the bonding effectiveness of some materials appears dramatically low, whereas the bonds of other materials are more stable (6,7).

A new class of Bonding agent has been introduced in which manufacturer claim that it can be used in Total etch and self-etch and selective etch mode (Tetric N Bond Universal vivapen, Single Bond Universal) the literature is replete in studies comparing this new class of bonding agents.

Hence the aim of this study is to evaluate the degree of bond strength produced by these new commercially available bonding agents [Tetric N Bond Universal Vivapen (IvoclarVivadent) and Single Bond Universal (3M ESPE)], and compare their bond strength produced by a total etch bonding system (Tetric N Bond ) and Self Etch (Clearfil SE).

Null Hypothesis: There is no difference in shear bond strength of the Tetric N Bond Universal Vivapen (Total Etch/Self Etch) ,Single Bond Universal, (Total Etch/Self Etch),Tetric N Bond (Total Etch), ClearFil SE(Self Etch).

## Material and Methods

96 freshly extracted non carious permanent human molars were included in this study. Carious, previously restored, fractured tooth and teeth with attrition, abrasion, erosion were excluded. The teeth were debrided by rinsing under running water followed by prophylaxis and polished with pumice rubber cups followed by storage in isotonic saline.

Each tooth was decoronated using a diamond disc with water coolant until dentin was exposed. The cut dentin surface was then abraded against 600-grit wet silicon carbide papers for 60 seconds to produce a uniform smear layer. The remaining apical part of each tooth up to 1mm from cemento- enamel junction was mounted on a plastic ring using acrylic resin. The teeth were randomized using computer generated random number tables into two groups of 48 teeth each and each group was sub divided into three sub groups of 16 each.

-Distribution of samples 

The teeth were randomly divided into two groups consisting of 48 teeth each.  Table 1 shows the distribution of teeth in to Group A and Group B which were sub divided in to three groups each.

**Table 1:**
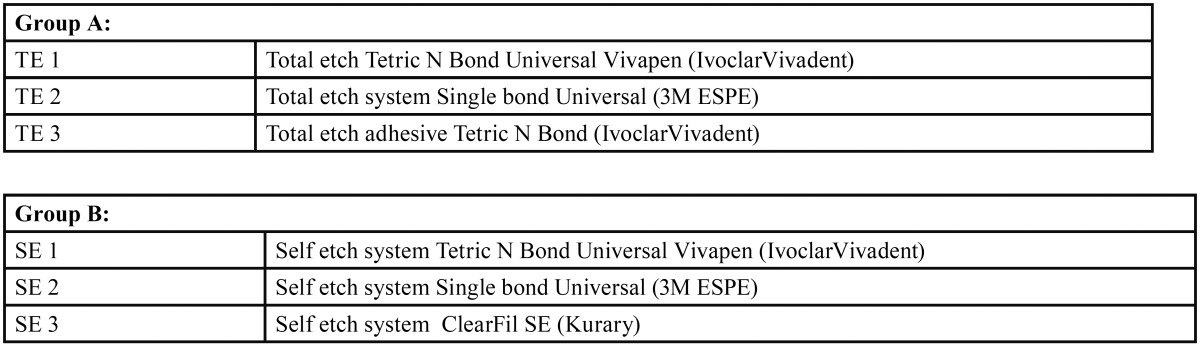
Division of group.

Application of dentin bonding system and resin composite:

The dentin bonding systems were applied following manufacturer’s instructions in all three sub-groups.

• Group A – TE TNB (Tetric N Bond Universal Vivapen), TE SBU (Single bond Universal),TE TNT (Tetric N Bond )

Etchant was applied on cut tooth surface and etched for 15 seconds followed by rinsing for 10 seconds. After drying excess water, 2-3 consecutive coats of adhesive was applied with gentle agitation and air thinned for 5 seconds. Followed by light curing for 10 seconds.

• Group B – SE TNB (Tetric N Bond Universal), SE SBU(Single bond Universal ) SE CSE ( ClearFil SE).

2-3 consecutuive coats of self etch adhesive were applied with gentle agitation and air thinned for 5 seconds. Followed by light curing for 10 seconds.

For all groups, the composite was placed in 2-3 layers using Teflon mold of dimensions 3mm in height and 2mm in diameter. Each increment was cured using a LED curing unit according to manufacturer’s instruction.

After 24-h storage in distilled water at 37ºC, the samples were thermocycled between 50ºC-55ºC with a dwell time of 5sec for 500 thermocycles.

The shear bond strength was determined using a knife-edge blade in a universal testing machine at a crosshead speed of 1 mm/min and readings were recorded in kgF. Data were subjected to statistical analysis using one-way analysis of variance (ANOVA) (*P*<0.05) and post hoc tukey’s test for inter and intra group analysis respectively.

## Results

The mean shear bond strength of specimens in group A (total-etch) showed higher bond strength value for Tetric N Bond total etch (TNT) followed by Single Bond Universal (SBU) and Tetric N Bond Universal (TNB) but statistically showed no significance between the groups ( Table 1. Graph A,C). Similarly specimens in group B(self-etch) showed higher bond strength value for Clearfil SE (CSE) followed by Single Bond Universal (SBU) and Tetric N Bond Universal (TNB) and showed statistically significant value between Clearfil SE (CSE) and Single Bond Universal (SBU), Tetric N Bond Universal (TNB) with *p* value <0.0001 ( Table 2, Table 3). Graph B,D) but showed no statistical significance between Single Bond Universal (SBU) and Tetric N Bond Universal (TNB) ( Table 4).

**Table 2:**
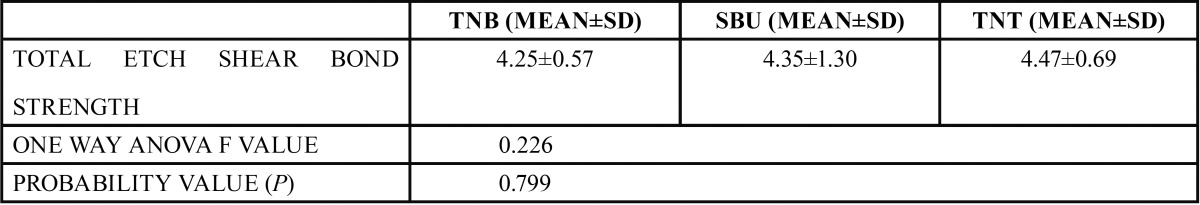
Shows comparison of One - way ANOVA analysis of samples under total etch.

**Table 3:**
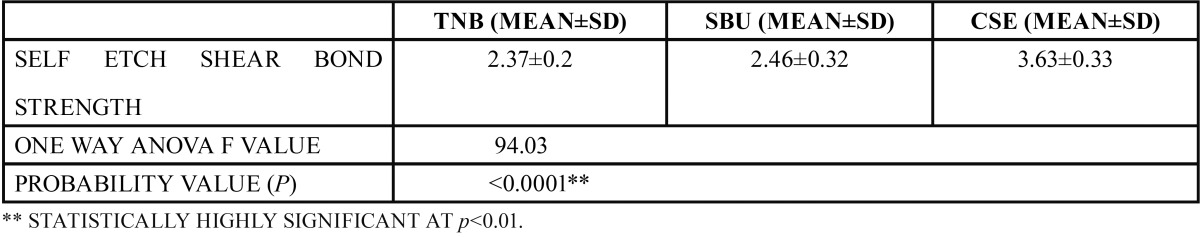
Comparision of One - way ANOVA analysis of samples under self-etch group.

**Table 4:**
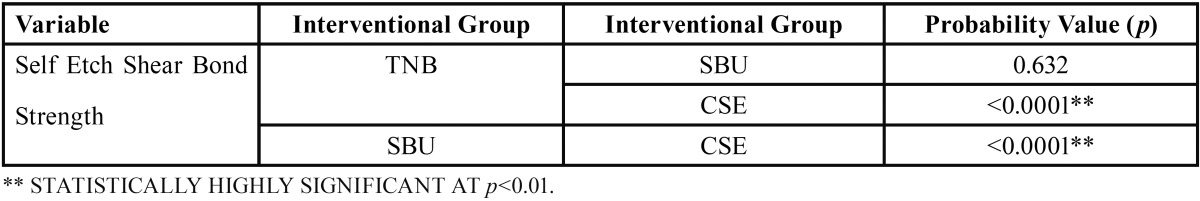
TUKEY’S POST HOC FOR TABLE 2.

## Discussion

Adhesion is defined as the mechanism that bonds two materials in intimate contact across an interface. The key element for adhesion is the intimacy of the bond that develops between the adhesive and the substrate. While effective adhesion to enamel is achieved with relative ease, adhesion to dentin poses a difficult challenge. This is partly due to the biological characteristics of dentin, namely its high organic content, its tubular structure, and the presence of the dentin smear layer that forms immediately after cavity preparation (3,8).

Thus for effective bonding smear layer can be totally removed as in total etch technique or it can be modified as in self etch technique which leads to hybridization at resin-dentine interface by a molecular level mixture of adhesive polymers and dentinal hard tissues (5).

Thus a new type of single-step self-etch adhesive that is categorizedas “universal” or “multi-mode” has been recently introduced for patient care. These bonding systems are recommended by dental manufacturers’ for use both with and without acid pretreatment of enamel surfaces. In order to overcome the lower bond strengths to enamel reported for self-etch adhesive systems, universal adhesives can be used with either etch-and-rinse or self-etch approaches (9).

However, this type of adhesive was only recently introduced to the market, and there is limited information as to whether the different etching modes achieve equivalent bonding performance to dentin when it is subjected to repeated sub-critical loading. Thus, the focus of this laboratory research investigation was to check and compare bond durability of a resin composite using newer universal adhesives with different etching modes on a single substrate, dentin.

Thus in the present study comparision of SBS of single bond universal (3MSPE) and tetric N bond universal (ivoclarvivadent) was done with tertic n bond total etch in total etch mode and with clear fill SE in self etch mode and no significant difference of universal bonding agents either in total etch group or self-etch group was found.

One of the keys of success with self-etching adhesives is the chemical bonding potential of their functional monomers to hydrox-yapatite (HAp),1 as described by the ‘‘adhesion/ decalcification concept’’. Among the currently used functional monomers, 10 methacryloyloxydecyldihydrogenphosphate (MDP) has demonstrated a very effective and durable bond to dentine, due to the low solubility of the calcium salt that forms on the hydroxyapatite surface. On the other hand, micromechanical interlocking by means of good dentine hybridization (i.e. resin tags and hybrid layer), has been proposed to improve the bond strength of SEAs. Phosphoric acid etching of dentine prior to application of SEAs significantly improves the interface infiltration morphology, by generating thicker hybrid layers and longer resin tags. Removal of the smear layer and smear plugs by this pre-treatment facilitates the adhesive penetration, especially in mild SEAs (9).

Tetric N-Bond Universal and universal adhesives usually, contain low levels of acidic monomer, and are therefore “mild-etching” adhesives. Tetric N-Bond Universal has a pH of approximately 2.5 – 3.0. The Tetric N-Bond Universal matrix is based on a combination of monomers of hydrophilic (hydroxyethyl methacrylate/HEMA), hydrophobic (decandioldimethacrylate/D3MA) and intermediate (bis-GMA) nature. This combination of properties allows Tetric N-Bond Universal to reliably bridge the gap between the hydrophilic tooth substrate and the hydrophobic resin restorative, under a variety of surface conditions.

Single Bond Universal has a pH of approximately 2.7. Chemical bonding in Single Bond Universal between 10-MDP and enamel/dentine may play an important role in forming stable and durable interfaces by providing acidity for its self-etch capability. The chemical bonding provided by the 10-MDP molecule in the primer was combined with the excellent mechanical properties and high conversion rate of its filled hydrophobic resin (10).

Thus the presence of MDP in the composition of SBU may explain the higher SBS. SBU also contains the polyalkenoic acid co-polymer (Vitrebond Copolymer), which in combination with MDP has shown contradictory results in the literature. The polyalkenoic acid copolymer may compete with the MDP monomer for Ca-bonding sites in HAp and due to its high molecular weight, could even prevent monomer approximation during polymerization.

Considering the short time elapsed since these new universal adhesives introduced in the market, only little clinical outcomes are available in the literature. A clinical evaluation of SBU under different application modes (self-etch or etch-and-rinse) in caries-free cervical restorations was performed by Perdigão *et al.* In their 18-month study, the adhesive showed a low incidence of clinical failures, regardless of the bonding strategy used. This data seems to correlate well with the results in the present study for the same material, as well as with previous *in vitro* results of the same author.

*In vitro* bond strength to dentin is influenced by several factors, such as the type and age of the teeth, the degree of dentin demineralization, and the bond of dentin surface, the type of bond strength test, the storage media and environmental surface humidity (11).

The clinical implication of this study is that universal adhesives might be used in both total etch and self-etch mode to dentine without a significant difference in bond strength. Therefore, more information is necessary to predict the long term bonding durability of universal adhesives.

## Conclusions

Within the limits of this study, we can conclude that application of an etching step prior to Universal Adhesives significantly improves their dentine penetration pattern, although this does not affect their mean SBS. The bond strength values of the TBU regardless of application mode were comparable to SBU making them reliable for working under different clinical conditions.
